# Metabolism of eriocitrin in the gut and its regulation on gut microbiota in mice

**DOI:** 10.3389/fmicb.2022.1111200

**Published:** 2023-01-12

**Authors:** Xia Meng, Hongchen Wu, Jiayi Xiong, Yongquan Li, Lin Chen, Qing Gu, Ping Li

**Affiliations:** Key Laboratory for Food Microbial Technology of Zhejiang Province, College of Food Science and Biotechnology, Zhejiang Gongshang University, Hangzhou, Jiangsu Province, China

**Keywords:** eriocitrin, metabolism, gut microbiota, short-chain fatty acids, dietary intervention

## Abstract

**Introduction:**

Eriocitrin, found in lemon fruit, has shown a wide range of biological properties. Herein, we investigated the intestinal metabolic profile of eriocitrin in colon, and the regulation of dietary intervention of eriocitrin on gut microbiota.

**Methods:**

We performed ultra performance liquid chromatography-electrospray ionization-tandem mass spectrometry (UPLC-ESI-MS/MS), 16S rDNA gene sequencing and gas chromatography-mass (GC-MS) on colon contents from the eriocitrin group (*n*=6), and compared them with control participants (*n*=6).

**Results:**

A total of 136 flavonoids were found in colon contents, including eriocitrin and its six metabolites (eriodictyol, homoeriodictyol, hesperetin, eriodictyol-3′-O-glucoside, hesperetin-7-O-glucoside and eriodictyol-7-O-(6″-O-galloyl) glucoside). Moreover, dietary intervention of eriocitrin significantly alters the beta diversity of the gut microbiota, the probiotics such as *Lachnospiraceae_UCG_006* were significantly enriched, and the production of butyrate, valerate and hexanoate in the colon pool of short-chain fatty acids were significant increased. The spearman’s association analysis performed some intestinal bacteria may be involved in the metabolism of eriocitrin.

**Discussion:**

Collectively, our results preliminarily suggest the metabolism of eriocitrin in the gut, demonstrating alterations of eriocitrin in gut microbiota, which warrants further investigation to determine its potential use in food and biomedical applications.

## 1. Introduction

Eriocitrin is a flavonoid widely existed in citrus fruits such as lemons (Miyake et al., 1997). Previous studies have demonstrated that eriocitrin has obvious lipid-lowering effects on both high-fat and high-cholesterol fed rats (Miyake et al., 2006) and DIO-zebrafish (Hiramitsu et al., 2014). It suppresses muscle atrophy by reducing oxidative stress in the skeletal muscles of mice (Takase et al., 2021). Besides, it can also effectively prevent the occurrence of diseases such as allergies, tumors and cancer *in vivo* (McKay and Blumberg, 2006; Wang et al., 2016).

Evidence is accumulating that the biological activity of eriocitrin is closely related to its metabolites. Pharmacokinetic studies have shown that multiple metabolites of eriocitrin were found in the blood, feces, urine (Li et al., 2019), and various organs (Ferreira et al., 2021) of rats after dietary intervention of eriocitrin, and eriodictyol, hesperetin, and homoeriodictyol have been identified as the main metabolites. Wang et al. (2016) demonstrated that eriocitrin can be metabolized into eriodictyol first, and then undergo phase II metabolism such as glucuronidation, sulfation, and methylation, thereby producing hesperetin, homoeriodictyol, eriodictyol-O-glucuronide, and other compounds (Nielsen et al., 2000; Ávila-Gálvez et al., 2021). Furthermore, an earlier study identified the products of eriocitrin metabolized by intestinal bacteria derived from the human gut *in vitro*, which examined eriocitrin can be metabolized to eriodictyol by gut bacteria such as *Bacteroides*, *Bifidobacterium*, and *Enterobacter* (Miyake et al., 1997). However, there have been few studies on the metabolism of eriocitrin *in vivo* by gut microbiota.

The gut microbiota is a complex biological community, which resides primarily in the colon of the digestive system of humans and mammals (Sekirov et al., 2010). Environment, diet, and age all have an impact on the establishment and stability of gut microbiota (Rinninella et al., 2019). Growing evidence suggests that the occurrence of some diseases, such as obesity (Ley et al., 2005), cancer (Garrett, 2015), and cardiovascular disease (Tang et al., 2017), is related to the imbalance of intestinal microbial.

Flavonoids are known to positively shape gut microbiota composition. Polymethoxyflavonoids (Zeng et al., 2020), hesperetin (Unno et al., 2015), hesperidin (Estruel-Amades et al., 2019), Isoorientin (Yuan et al., 2018), and a variety of extracted dietary flavonoids can change the structure of intestinal microbiota, which is manifested as an increase in beneficial bacteria and a decrease in harmful bacteria (Li et al., 2023). But the effects of eriocitrin dietary interventions on the structure and activity of the gut microbial community *in vivo* has yet to be realized.

The present study focused on the metabolism of eriocitrin in the colon and its regulation on gut microbiota. For this purpose, the eriocitrin metabolites, gut microbiota and short-chain fatty acids in mice colonic contents were analyzed. Afterwards, the spearman correlation analysis between eriocitrin metabolites and intestinal bacteria was performed to explore the role of different intestinal bacteria in the metabolism of eriocitrin *in vivo*.

## 2. Materials and methods

### 2.1. Materials and animals

Six-week-old male Institute of Cancer Research (ICR) mice (*n* = 12) were from Zhejiang Center of Laboratory Animals. The mice were randomly divided into eriocitrin treatment and control group (6 males per group), were maintained under standard conditions (temperature 23 ± 2°C, 12-h light/dark cycle, 50 ± 10% humidity) with free access to food and drinking water for 1 week prior to experiment. Mice in the eriocitrin (Yuanye, Shanghai, and China) group were given eriocitrin by gavage, which was dissolved in 0.9% saline at a dose of 100 mg·kg^−1^·d^−1^. However, the control group mice were given the same dose of 0.9% saline. After 2-weeks of feeding, mice were abrosiaed with CO_2_ asphyxiation after 12 h fasting, and colon faeces of mice were collected and stored at −80°C until later microbial and metabolite analysis. The animal study was reviewed and approved by Zhejiang Center of Laboratory Animals (2022R0005).

### 2.2. Metabolite of flavonoids extraction and analysis

#### 2.2.1. Sample preparation and extraction

Colon contents were removed from −80°C refrigerator and thawed on ice, then vortex for 10 s. Mix 50 mg of sample and 1.2 mL of 70% methanol internal standard extract, scroll for 15 min. Centrifuged (12,000 r/min, 4°C) for 3 min, Then the supernatant was filtered with a microporous filter mem-brane (0.22 μm) and stored in a sample flask for LC–MS/MS test.

#### 2.2.2. Identification of flavonoids

The sample extracts were analyzed using an ultra performance liquid chromatography-electrospray ionization-tandem mass spectrometry (UPLC-ESI-MS/MS) system (UPLC, SHIMADZU Nexera X2; MS, Applied Biosystems 4,500 Q TRAP, US). Chromatographic Separation with an Agilent SB-C18 column (1.8 μm, 2.1 mm * 100 mm) maintained at 40°C and eluted at a flow rate of 0.3 mL/min. The mobile phase was consisted of solvent A (pure water with 0.1% formic acid) / solvent B (acetonitrile with 0.1% formic acid), initially composed of 95:5 (v/v), then linearly decreased mobile phase A to 5:95 in 9 min and held for 1 min, and then increased mobile phase A content to 95:5 for 5 min. The injection volume was 4 μL, and the effluent was alternatively connected to an ESI-triple quadrupole-linearion trap (QTRAP)-MS.

Linear ion trap (LIT) and triple quadrupole (QQQ) scans were acquired on a triple quadrupole-linear ion trap mass spectrometer (QTRAP), AB4500 QTRAP UPLC/MS/MS System, equipped with an electrospray ionization (ESI) Turbo Ion-Spray interface, operating in positive and negative ion mode and controlled by Analyst 1.6.3 software (AB Sciex). The ESI source operation parameters were as follows: ion source, turbo spray; source temperature 550°C; ion spray voltage (IS) 5,500 V (positive ion mode)/−4,500 V (negative ion mode); ion source gas I (GSI), gas II(GSII), curtain gas (CUR) were set at 50, 60, and 25.0 psi, respectively; the collision-activated dissociation(CAD) was high. Instrument tuning and mass calibration were performed with 10 and 100 μmol/L polypropylene glycol solutions in QQQ and LIT modes, respectively. QQQ scans were acquired as multiple reaction monitoring (MRM) experiments with collision gas (nitrogen) set to medium. Decluttering potential (DP) and collision energy (CE) for individual MRM transitions was done with further DP and CE optimization. A specific set of MRM transitions were monitored for each period according to the metabolites eluted within this period (Chen et al., 2013).

### 2.3. DNA extraction and 16S rDNA sequencing of colon contents from mice

DNA from samples of colon contents was extracted using CTAB according to manufacturer’s instructions. The total DNA was eluted in 50 μL of Elution buffer and storedat −80°C until measurement in the PCR by LC-Bio Tech Co., Ltd. (Zhejian, China).

Samples were sequenced on an Illumina NovaSeq platform, and Paired-end reads were merged using FLASH. Quality filtering on the raw reads was performed under specific filtering conditions to obtain high-quality clean tags according to fqtrim (v0.94). Chimeric sequences were filtered using Vsearch software (v2.3.4). After dereplication using divisive amplicon denoising algorithm (DADA2), we obtained the feature table and feature sequence (Callahan et al., 2016).

### 2.4. Colon contents short chain fatty acids (SCFAs) analysis

The colon content samples (0.1 g) were mixed with 0.5 mL of water, 200 μL of 50% sulfuric acid, 50 μL of cyclohexanone and 1 mL of ether and homogenized for 1 min. Centrifuged (12,000 r/min, 4°C) for 3 min to get the supernatant. SCFA profiles were measured by gas chromatography–mass spectroscopy (GC–MS QP2010-Ultra, Shimadzu, Kyoto, Japan) equipped with an Agilent DB-WAX column (30 m*0.25 mm*0.25 μm) at a flow rate of 1 mL/min, and cyclohexanone was used as the internal standard. The injection port, column, and detector were maintained, respectively, at 220, 230, and 220°C, and the temperature elution ramp was: 60°C for 1 min initially, then shifted to 210°C for 3 min.

### 2.5. Data handling and statistical analysis

Six biological replicates were performed in each experiment. The principal component analysis (PCA) of flavonoid metabolites were carried out using R (Thévenot et al., 2015). Alpha diversity and beta diversity were calculated by QIIME2 (Bolyen et al., 2019). Datas were analyzed using GraphPad Prism8, and expressed as means ± SD. The significant difference among groups was tested using student test. Differences were considered significant at *p* < 0.05.

## 3. Results

### 3.1. Identification of flavonoid metabolites in colon content

Comparing the body weight, liver and spleen index of the mice in the eriocitrin group and the control group, no observed difference was found between the groups (*p* > 0.05; Supplementary Table S1). Indicating that 100 mg·kg^−1^·d^−1^ eriocitrin in diet had no appreciable toxic effect on mice.

To explore eriocitrin metabolites in mice colon, flavonoid metabolites in mice colon contents were identified by UPLC-ESI-MS/MS. A total of 136 flavonoids were identified by widely targeted metabolomics (Supplementary Table S2). Among them, we found eriocitrin and its six flavonoid metabolites, including eriodictyol, hesperetin, homoeriodictyol, eriodictyol-3′-O-glucoside, hesperetin-7-O-glucoside, and eriodictyol-7-O-(6″-O-galloyl) glucoside (Table 1). A number of unexpected flavonoids were detected, most likely because there were many dietary flavonoid residues in the mice.

**Table 1:** Eriocitrin metabolites in mice colonic contents.

Compounds	RT (min)	Q1 (Da)	Q3 (Da)	Formula
Eriocitrin	3.58	595.17	287.06	C_27_H_32_O_15_
Eriodictyol	5.03	287.06	135.00	C_15_H_12_O_6_
Eriodictyol-3′-O-glucoside	4.27	449.11	287.06	C_21_H_22_O_11_
Hesperetin-7-O-glucoside	4.33	465.14	303.09	C_22_H_24_O_11_
Hesperetin	5.63	303.09	177.05	C_16_H_14_O_6_
Homoeriodictyol	5.55	303.09	153.02	C_16_H_14_O_6_
Eriodictyol-7-O-(6″-O-galloyl)glucoside	4.09	601.12	439.09	C_28_H_26_O_15_

Q1 (Da): the molecular weight of the parent ion after adding ions by the electrospray ion source; Q3 (Da): tolecular weight of characteristic fragment ions.

**Figure 1:**
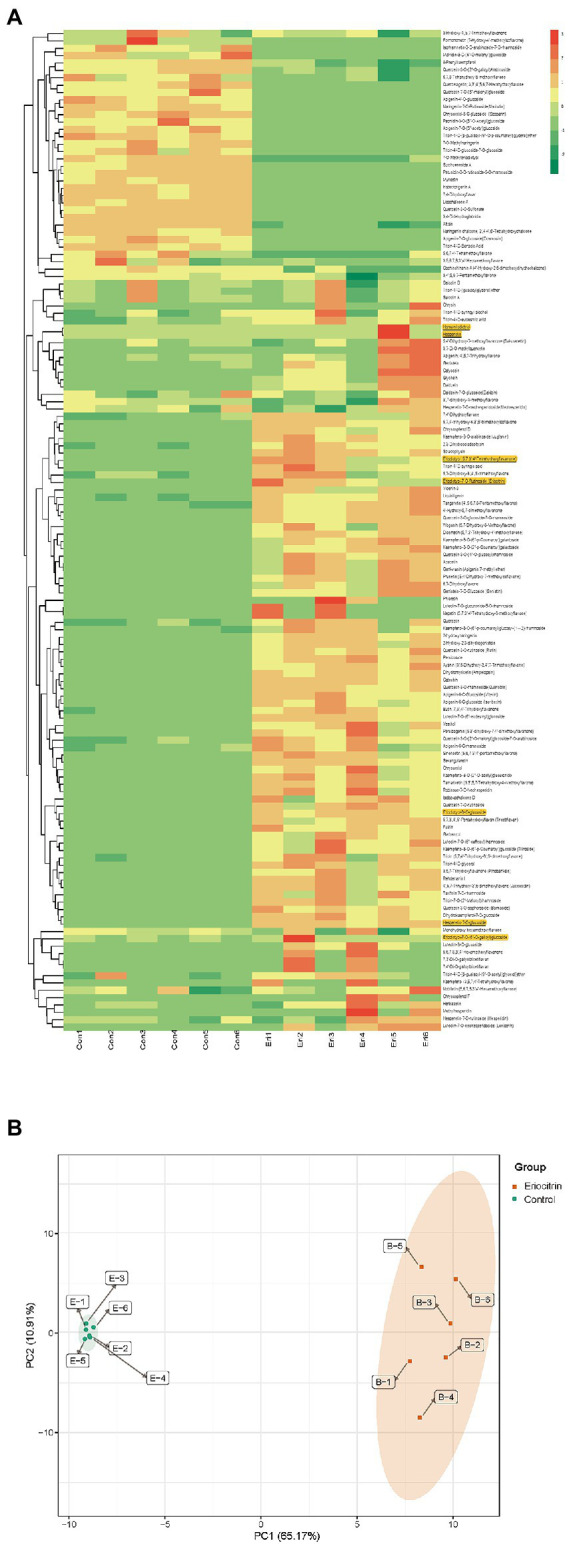
Differential flavonoid metabolite analysis on the basis of clustering heatmap and principal component analysis (PCA). **(A)** Clustering heatmap of all flavonoid metabolite, the metabolite content data was normalized by maximum difference normalization method. Each sample is visualized by a separate column and each metabolite is represented by a separate row, up-regulated and down-regulated metabolites are expressed in shades of red and green, respectively. **(B)** PCA score plot. Eir, eriocitrin; Con, control.

All flavonoid metabolites of the samples are shown as a heatmap after homogenization (Figure 1A), by clustering all flavonoid metabolites, nearly two-thirds of the flavonoid metabolites content in eriocitrin group was higher than those in control group. PCA reflects the characteristics of metabolomics multidimensional data through several principal components. In this study, the PCA results showed that between-group samples are clearly separated, and the repeated samples were compactly gathered together (Figure 1B). In conclusion, the contents of flavonoid metabolites in the eriocitrin group and the control group were significantly different, and the experiment was reproducible and reliable.

### 3.2. Differential metabolite analysis.

**Figure 2:**
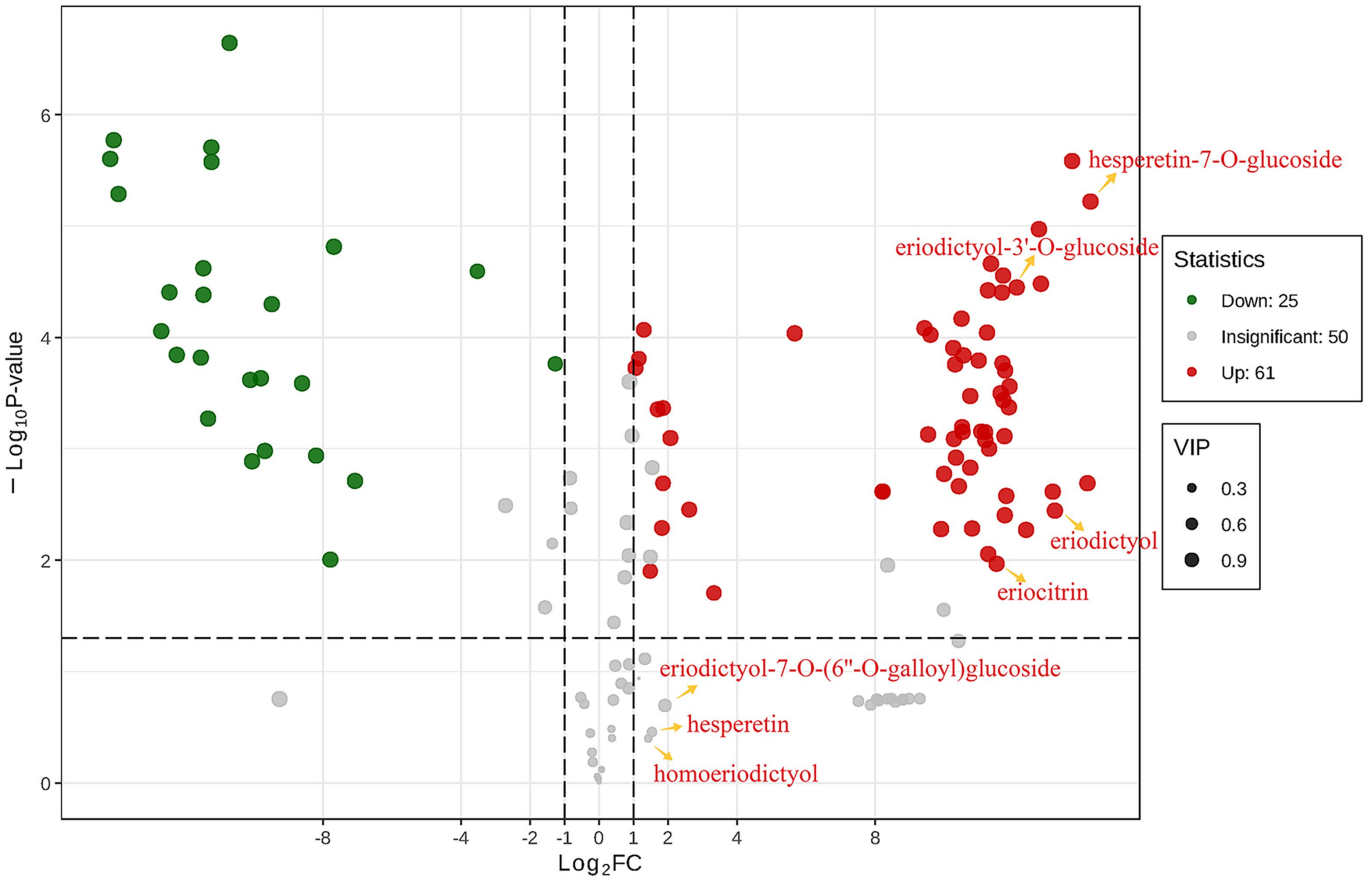
Differential metabolites analyses for control group vs. eriocitrin group by criteria set at FC ≥ 2 or ≤ 0.5, VIP ≥ 1 and *p* < 0.05. each point in the figure represents a metabolite, where red and green points represent up-regulated and down-regulated differential metabolites, respectively, and gray points represent metabolites that were detected but not significantly different. The abscissa represents the logarithm of the relative content difference of a metabolite in the two groups of samples (log_2_FC), the ordinate represents the significance level of the difference (−log10P-value), and the size of the dot represents the VIP value.

Further finding out the content changes of eriocitrin metabolites by differential metabolite analysis. Differential metabolites were screened by fold change (FC), variable importance in projection (VIP) and *p*-value, when metabolites satisfy FC ≥ 2 or ≤ 0.5, VIP ≥ 1 and *p* < 0.05, they are considered to be differential metabolites. There were 86 differential flavonoid metabolites between the two groups (Supplementary Table S3), 61 metabolites were up-regulated and 25 were down-regulated compared to control group (Figure 2). Among them, the contents of eriocitrin, eriodictyol, eriodictyol-3′-O-glucoside and hesperetin-7-O-glucoside were up-regulated, while hesperetin, homoeriodictyol and eriodictyol-7-O-(6″-O-galloyl) glucoside did not change significantly. However, the FC values also indicated that the content of hesperetin, homoeriodictyol and eriodictyol-7-O-(6″-O-galloyl) glucoside in the eriocitrin group was higher than that in the control group.

### 3.3. Effects of eriocitrin on gut microbiato in mice

To investigate the changes to the mice gut microbiota generated by dietary eriocitrin intervention, 16 s rDNA sequencing of colonic contents of six mice randomly selected from each group. Alpha diversity results showed that there were no significant changes in OTU richness, Chao1, Simpson and Shannon indices between the eriocitrin group and the control group (*p* > 0.05; Supplementary Table S4).

**Figure 3:**
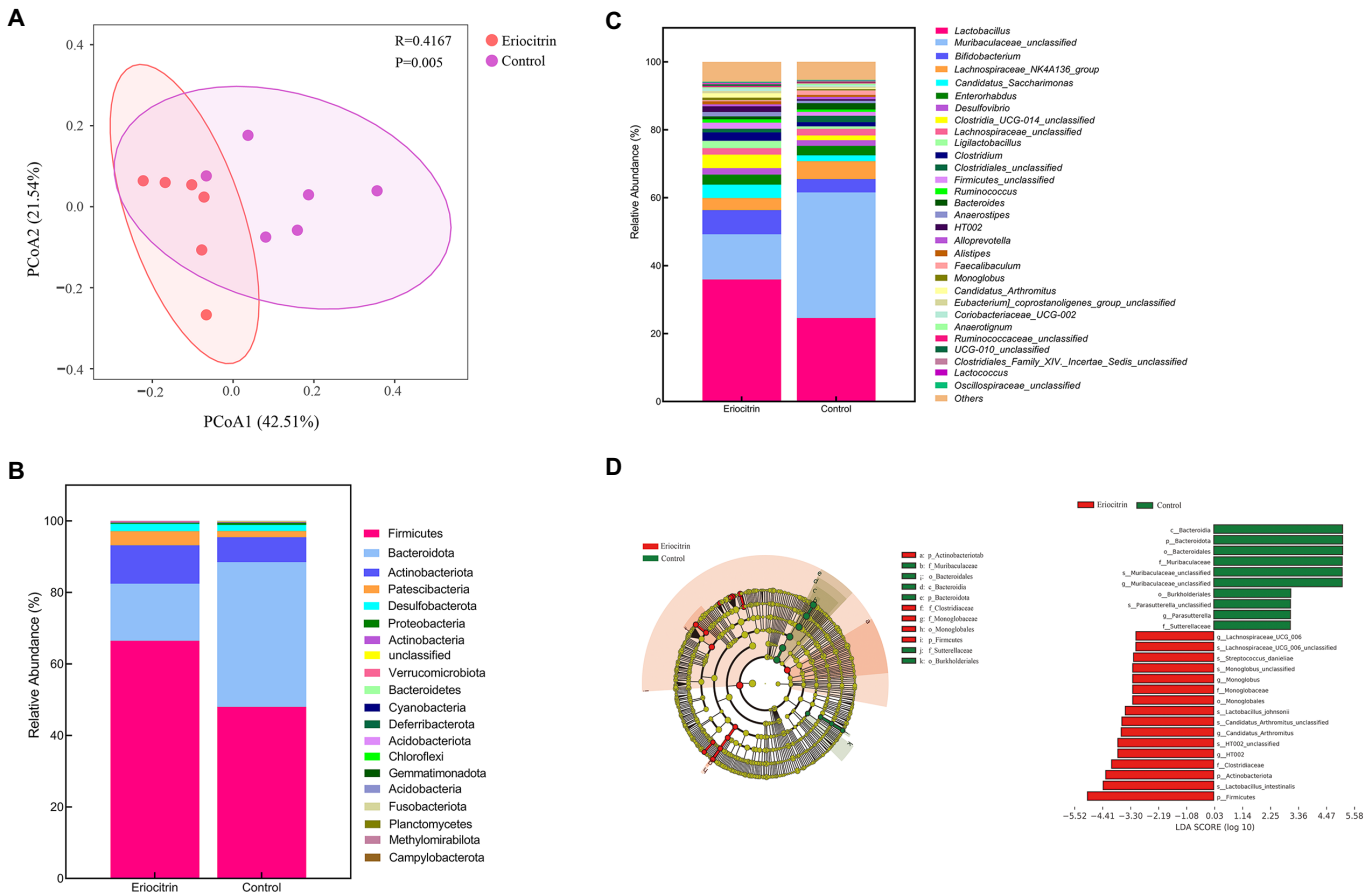
Dietary intervention with eriocitrin significantly alters the gut microbiota in mice. **(A)** Principal coordinates analysis (PCOA) based on UniFrac distance, each point represents a mouse sample. **(B)** Compositional change at the phylum level (*n* = 6). **(C)** Compositional change at the genus level (*n* = 6). **(D)** Gut microbiota genera differentially represented between eriocitrin and control groups (LDA > 3, *p* < 0.05).

Beta diversity was calculated using weighted UniFrac distances (Lozupone et al., 2011). The principal coordinates analysis (PCOA) of the weighted UniFrac distance performed on the microbial community structure of the eriocitrin group and the control group was significantly separated (Figure 3A), which proved that the eriocitrin had a significant effect on the gut microbiota of mice (*p* < 0.05).

We presented the relative abundance of the samples as a stacked bar graph. At the phylum level (Figure 3B; Supplementary Figure S1A), Firmicutes, Bacteroidetes and Actinobacteriota accounted for more than 90% of the total, and compared with the control group, Firmicutes and Actinobacteriota increased in the eriocitrin group, while Bacteroidetes decreased. At the genus level (Figure 3C; Supplementary Figure S1B), the top five dominant genera were *Lactobacillus*, *Muribaculaceae_unclassified*, *Bifidobacterium*, *Lachnospiraceae_NK4A136_group* and *Candidatus_Saccharimonas*. The specific differences in the intestinal bacteria of the two groups of samples are further illustrated by line discriminant analysis effect size (LefSe).

The LefSe analysis highlighted the differences in relative microbial abundance from phylum to species. All characteristic species were detected by Kruskal–Wallis, and the species with significant differences were obtained by detecting the difference in species abundance between different groups, and linear discriminant analysis (LDA) was used to obtain the final differential species with LDA > 3.0 (Figure 3D). Compared with the control group, Firmicutes (*p* = 0.025) and Actinobacteria (*p* = 0.025) were significantly increased, while Bacteroidetes (*p* = 0.004) were significantly decreased in the eriocitrin group. At the genus level, *Muribaculaceae_unclassified* (*p* = 0.004), *Muribaculum* (*p* = 0.037) and *Parasutterella* (*p* = 0.004) decreased significantly, while *Lachnospiraceae_UCG_006* (*p* = 0.007), *Monoglobus* (*p* = 0.025), *Candidatus_Arthromitus* (*p* = 0.016), *Lachnospiraceae_NK4B4_group* (*p* = 0.022), *Anaerofustis* (*p* = 0.049), *Faecalibacterium* (*p* = 0.002), *Gardnerella* (*p* = 0.007), and *HT002* (*p* = 0.007) increased significantly. At the species level, compared with the control group, some beneficial bacteria such as *Lachnospiraceae_UCG_006_unclassified*, *Streptococcus_danieliae*, *Lactobacillus_johnsonii*, *Lactobacillus_intestinalis*, and *Candidatus_Arthromitus_unclassified* increased, which further verified the probiotic function of eriocitrin.

### 3.4. Effects of eriocitrin on the content of SCFAs

**Figure 4:**
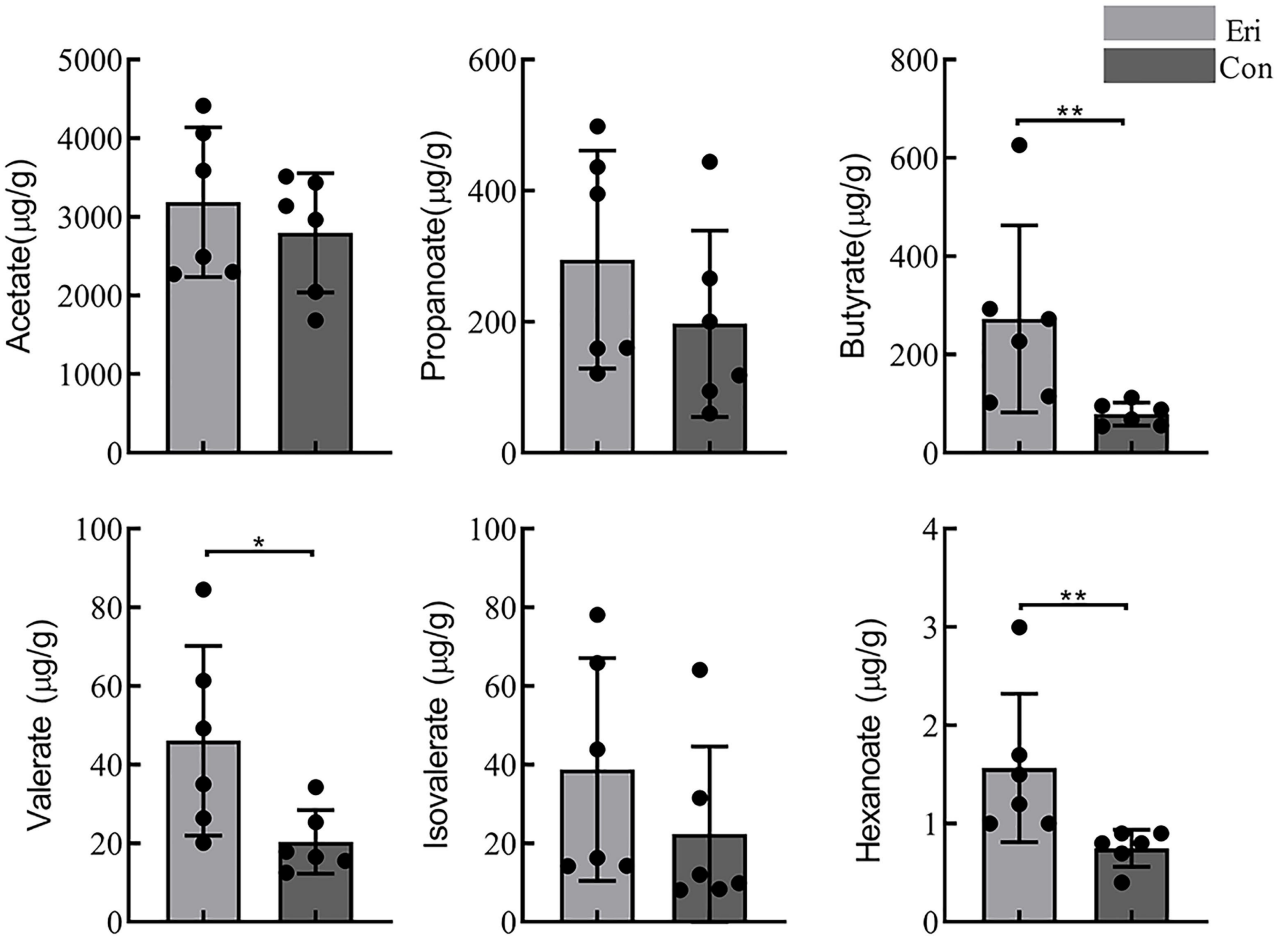
Short chain fatty acids content (SCFAs) in control and eriocitrin-treated mice colons. *p< 0.05, **p< 0.01.

Some carbohydrates that are difficult to be catabolized by the small intestine can be hydrolyzed by the gut microbiota to produce SCFAs (Cummings et al., 1987). It is commonly understood that the growth of pathogenic microorganisms could be inhibited by reducing luminal pH with more SCFA productions, eventually increasing the growth and activity of beneficial bacteria (Brownawell et al., 2012). The content of SCFAs in mice colon contents was detected by GC–MS. The results showed that acetate is the predominant SCFA in mice colon, and eriocitrin intervention diet can significantly increase the content of butyrate (*p* = 0.004), valerate (*p* = 0.015) and hexanoate (*p* = 0.002). However, no statistical difference was observed in content of acetate, propanoate and isovalerate between eriocitrin-administered mice and control mice (Figure 4).

### 3.5. Correlation analysis of gut microbiota and metabolites

Evidence is accumulating that gut microbiota population plays an essential and irreplaceable role in the *in vivo* metabolism of flavonoids, including deglycosylation of C-/O-glycosides, reduction of double bonds and cleavage of C-rings and demethylation of polymethoxyflavones. (Ian, 2018) Flavonoids metabolized by bacteria can be absorbed directly by the colon on one side, and on the other can be involved in blood circulation or excreted through urine (Wang et al., 2021). As with the previous results, we detected eriocitrin and its six metabolites (eriodictyol, homoeriodictyol, hesperetin, eriodictyol-3′-O-glucoside, hesperetin-7-O-glucoside, and eriodictyol-7-O-(6″-O-galloyl) glucoside) in the colon contents of mice. Indicating that eriocitrin can reach the mice colon intact and be metabolized into various metabolites.

**Figure 5:**
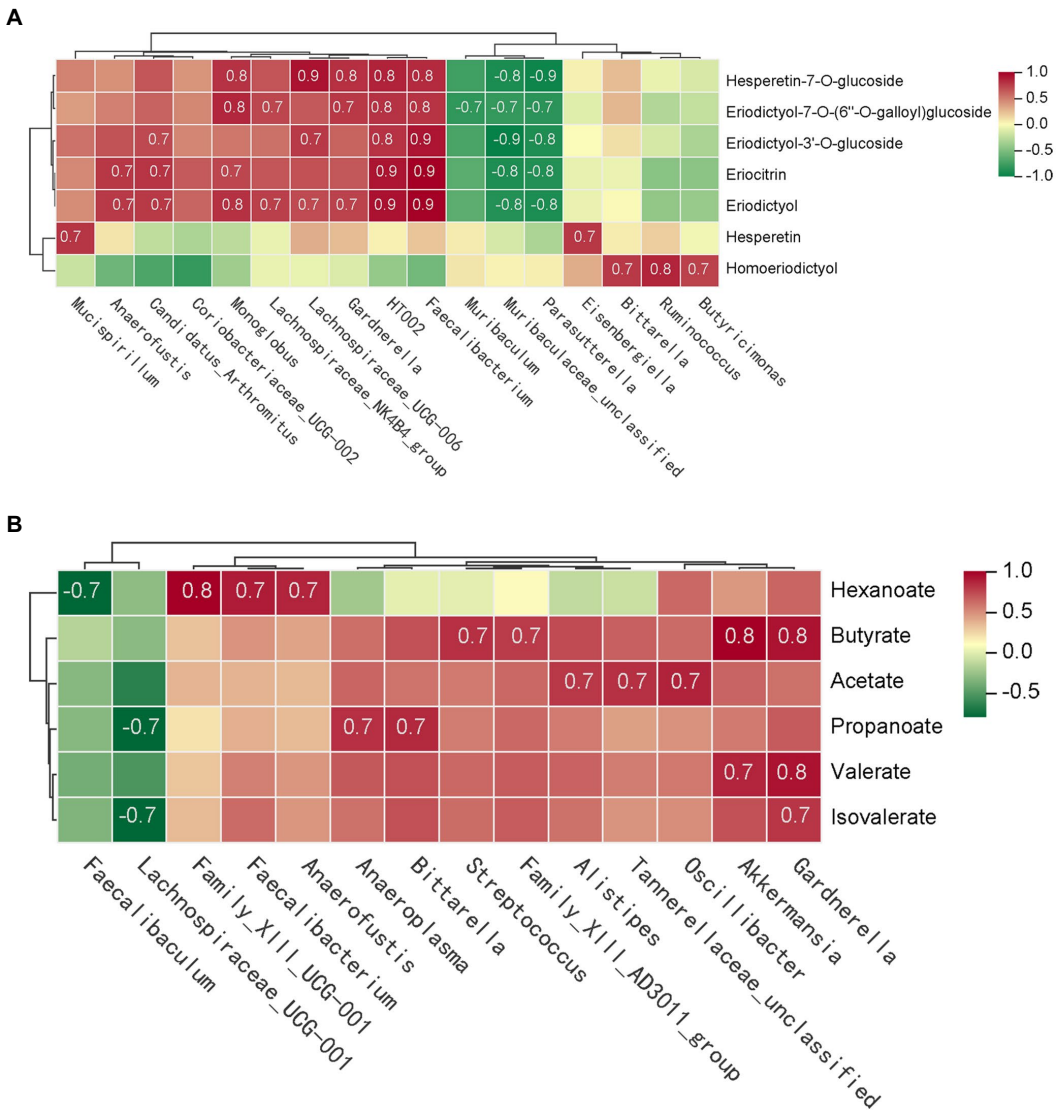
Correlation analysis of gut microbiota and metabolites. The matrices were derived from euclidean distance-based bi-clustering of spearman’s rank correlation matrices. Correlation coefficients in each square represent positive (red) and negative (blue) relationships. Colors are proportional to the absolute value of spearman’s rank correlations. **(A)** Correlation heatmap of gut microbiota and eriocitrin metabolites. **(B)** Heatmap of correlation coefficients between SCFAs production and gut microbiota.

In order to clearly identify the role of different gut bacteria in the metabolic of eriocitrin *in vivo*. We performed spearman’s correlation analysis between the eriocitrin metabolites and genus. And substances with spearman’s rank correlation coefficient |*r*| > 0.7 and *p* < 0.05 were selected for analysis. The results showed that *Monoglobus*, *Faecalibacterium*, *Candidatus_Arthromitus*, *Lachnospiraceae_UCG-006*, *Gardnerella*, and *HT002* were significantly positive correlation with most of the eriocitrin metabolites, and *Parasutterella* and *Muribaculaceae_unclassified* significantly negative correlations with them. In addition, hesperetin was positively correlated with *Mucispirillum* and *Eisenbergiella*, and we can also find that homoeriodictyol was positively correlated with *Butyricimonas*, *Ruminococcus* and *Bittarella* (Figure 5A).

Furthermore, spearman’s correlation analysis was performed to investigate the differences between microbial and SCFAs production (Figure 5B). Both butyrate and valerate were positively correlated with *Gardnerella* and *Akkermansia*, and hexanoate was positively correlated with *Anaerostipes*, *Faecalibacterium* and *Family_XIII_UCG-001*. Our study also found that acetate is closely related to several bacterial, such as *Oscillibacter*, *Alistipes* and *Tannerellaceae_unclassified*. However, almost six SCFAs were negatively correlated with *Lachnospiraceae_UCG-001* and *Faecalibaculum*.

## 4. Discussion

Several studies are currently being conducted to investigate the *in vivo* metabolism of eriocitrin, with the majority of them focusing on its metabolites in urine, blood, kidneys, liver and other organs. Nevertheless, few studies have been carried out on the rich intestinal metabolism of gut microbiota in the colon. The intestinal microbiota is a giant and complicated collection, and some gut bacteria have exhibited the ability to metabolize flavonoids, such as *Bifidobacterium*, *Bacteroides*, *Propionibacterium*, *Lactobacillus*, *Clostridium*, and *Eubacterium* (Miyake et al., 1997; Selma et al., 2009; Pereira-Caro et al., 2018). We detected a total of 136 flavonoids in the colonic contents of mice, of which 87 metabolites were significantly different, among these metabolites, we found eriocitrin and its six metabolites (eriodictyol, homoeriodictyol, hesperetin, Eriodictyol-3′-O-glucoside, Hesperetin-7-O-glucoside and Eriodictyol-7-O-(6″-O-galloyl) glucoside), which was higher in the eriocitrin group than that in the control group. A number of unexpected flavonoids were detected, most likely because there were many dietary flavonoid residues in the mice, and at the same time they were metabolized and partially absorbed.

Flavonoids hydrolyzed and metabolized by intestinal bacteria have a simpler structure, a lower molecular weight, and are easier to absorb by the body, allowing them to perform their functions more effectively (Blaut et al., 2003). Some flavanone catabolites, have been proven to have greater biological activity than the precursor flavanones (Kay et al., 2017; Murota et al., 2018). Taking antioxidant capacity as an example, in our experiment, eriodictyol and hesperetin were detected as eriocitrin metabolites, and studies have shown that their anti-lipid peroxidation capacity is significantly better than that of eriocitrin (Miyake et al., 1997, 2000).

Our results indicated that eriocitrin-inthatvention diet alters the beta diversity of the gut microbiota in mice. At the phylum level, the relative abundance of Firmicutes and Actinobacteriota in mice gut was significantly higher in the eriocitrin treatment group, while Bacteroidetes were significantly reduced. The specific differences between the two groups of samples were then analyzed by LefSe, and it was found that eriocitrin could significantly reduce the abundance of *Muribaculaceae_unclassifie* and *Parasutterella*, and significantly increase the abundance of *Lachnospiraceae_UCG_006*, *Monoglobus*, *Candidatus_Arthromitu*, *Lachnospiraceae_NK4B4_group*, *Anaerofustis*, *Faecalibacterium*, *Gardnerella*, and *HT002*. Several studies demonstrated that *Parasutterella* is not only related to systemic metabolic abnormalities in rats (Henneke et al., 2022), but also positively related to the occurrence of irritable bowel syndrome (Chen et al., 2018). A recent study found that *Parasutterella* may also be closely related to obesity (Henneke et al., 2022). *Lachnospiraceae UCG-006* is the most typical member of the family Lachnospiraceae, which are reputed to be SCFAs-producing bacteria (Flint et al., 2012). Noteworthy, momordica charantia fruit dramatically promoted the abundance of *Lachnospiraceae UCG-006*, and its blood lipid-lowering effect can be exerted (Zhang et al., 2020). A previous report showed that *Lachnospiraceae_UCG_006* is also associated with the treatment of lung cancer (Chen et al., 2022). All these evidences indirectly prove that the structural stability of gut microbiota can be improved by dietary intervention of eriocitrin.

**Figure 6:**
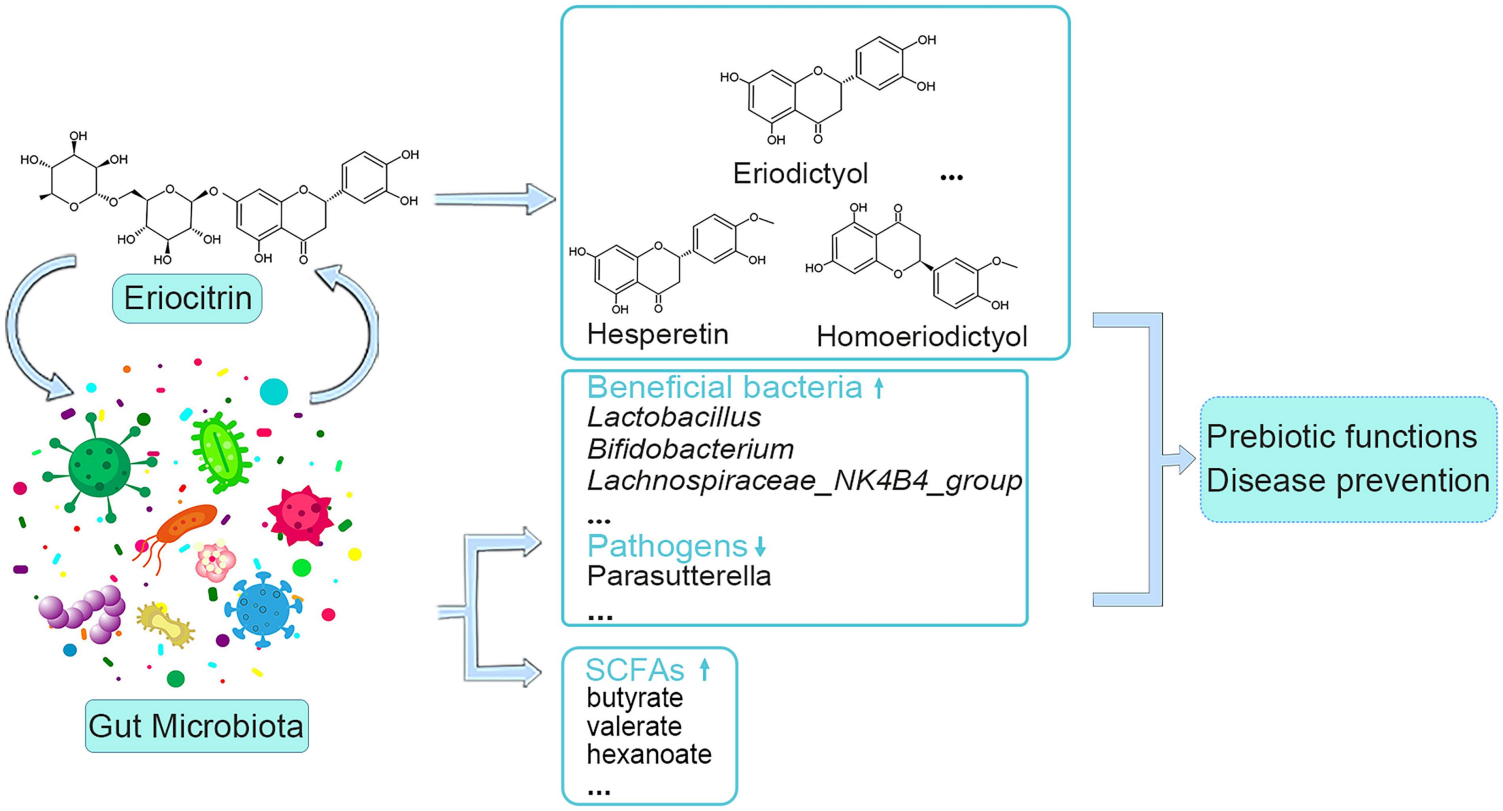
Schematic of interactions between eriocitrin and the gut microbiota.

SCFAs, derived from bacteria-dependent hydrolysis of fibers, and are the major components in regulating gut health (Tan et al., 2014). This study showed that eriocitrin can increase the content of all SCFAs in the colon of mice, butyrate, valerate and hexanoate had the most amplification among SCFAs. Butyrate plays an important role in maintaining intestinal barrier stability (Leonel and Alvarez-Leite, 2012), and it can also prevent and treat diet-induced obesity (Gao et al., 2009). Valerate lowers arterial blood pressure in rats (Onyszkiewicz et al., 2020). In addition, through correlation analysis, we found that bacteria such as *Gardnerella*, *Akkermansia*, *Absiella*, *Oscillibacter*, and *Ruminococcus* are associated with the production of SCFAs, which is consistent with previous research (Li et al., 2022). Spearman’s correlation analysis of differential microbiota and metabolites was carried out, and it was found that intestinal microbiota was closely related to eriocitrin metabolites and SCFAs. Therefore, we envision a circular causal diagram to explain the complex relationship between gut microbiota and eriocitrin metabolic homeostasis (Figure 6). Specifically, part of the eriocitrin reached the mice colon intact through the digestive tract. Eriocitrin undergoes processes such as demethylation and glucoglycation under the action of the intestinal flora and is catabolized into metabolites such as eriodictyol, hesperetin and homoeriodictyol, which may be related to the action of enzymes produced by some intestinal bacteria (Pereira-Caro et al., 2018). Correspondingly, the intestinal microecology is altered under the action of eriocitrin, on the one hand, the proportion of some bacteria associated with the metabolism of eriocitrin such as *Monoglobus*, *Faecalibacterium*, *Candidatus_Arthromitus*, and *Lachnospiraceae_UCG-006* increases, on the other hand, changes in the ratio of *Oscillibacter*, *Gardnerella*, *Akkermansia*, *Absiella*, and *Ruminococcus* promote the production of SCFAs. However, further microbiota transplantation experiments are needed to confirm the specific necessary correlation.

In conclusion, dietary intervention with eriocitrin alters flavonoid metabolites in the colon contents of mice, while also altering the structure of the gut microbiota, increasing the content of SCFAs, and association analysis was used to further demonstrate the interaction between eriocitrin and gut microbiota. However, this experiment also has certain limitations. Due to the interference of mice feed, the types of flavonoids identified are messy. In addition, the accurate determination of the metabolites of eriocitrin and its targeting function in the intestinal merit further investigation and verification. Our experiments provide valuable insights into eriocitrin research and application.

## Data availability statement

The data presented in the study are deposited in the NCBI’s Sequence Read Archive (SRA) repository, accession number GSE218729.

## Ethic statement

The animal study was reviewed and approved by Zhejiang Center of Laboratory Animals.

## Author contributions

PL and QG: conceptualization and supervision. XM and HW: methodology, investigation, and data curation. XM: writing-original draft preparation. JX, YL, and LC: writing-review and editing. PL: funding acquisition. All authors contributed to the article and approved the submitted version.

## Funding

This project was funded by National Natural Science Foundation of China (1110KZ0119614) and National Key Research and Development Program of China (2017YFE0122300).

## Conflict of interest

The authors declare that the research was conducted in the absence of any commercial or financial relationships that could be construed as a potential conflict of interest.

## Publisher’s note

All claims expressed in this article are solely those of the authors and do not necessarily represent those of their affiliated organizations, or those of the publisher, the editors and the reviewers. Any product that may be evaluated in this article, or claim that may be made by its manufacturer, is not guaranteed or endorsed by the publisher.
