# Supplementary figures and images for: Metabolism of eriocitrin in the gut and its regulation on gut microbiota in mice
Source: Front Microbiol. 2023 Jan 12;13:1111200. doi: 10.3389/fmicb.2022.1111200 (PMC9877458; doi:10.3389/fmicb.2022.1111200)

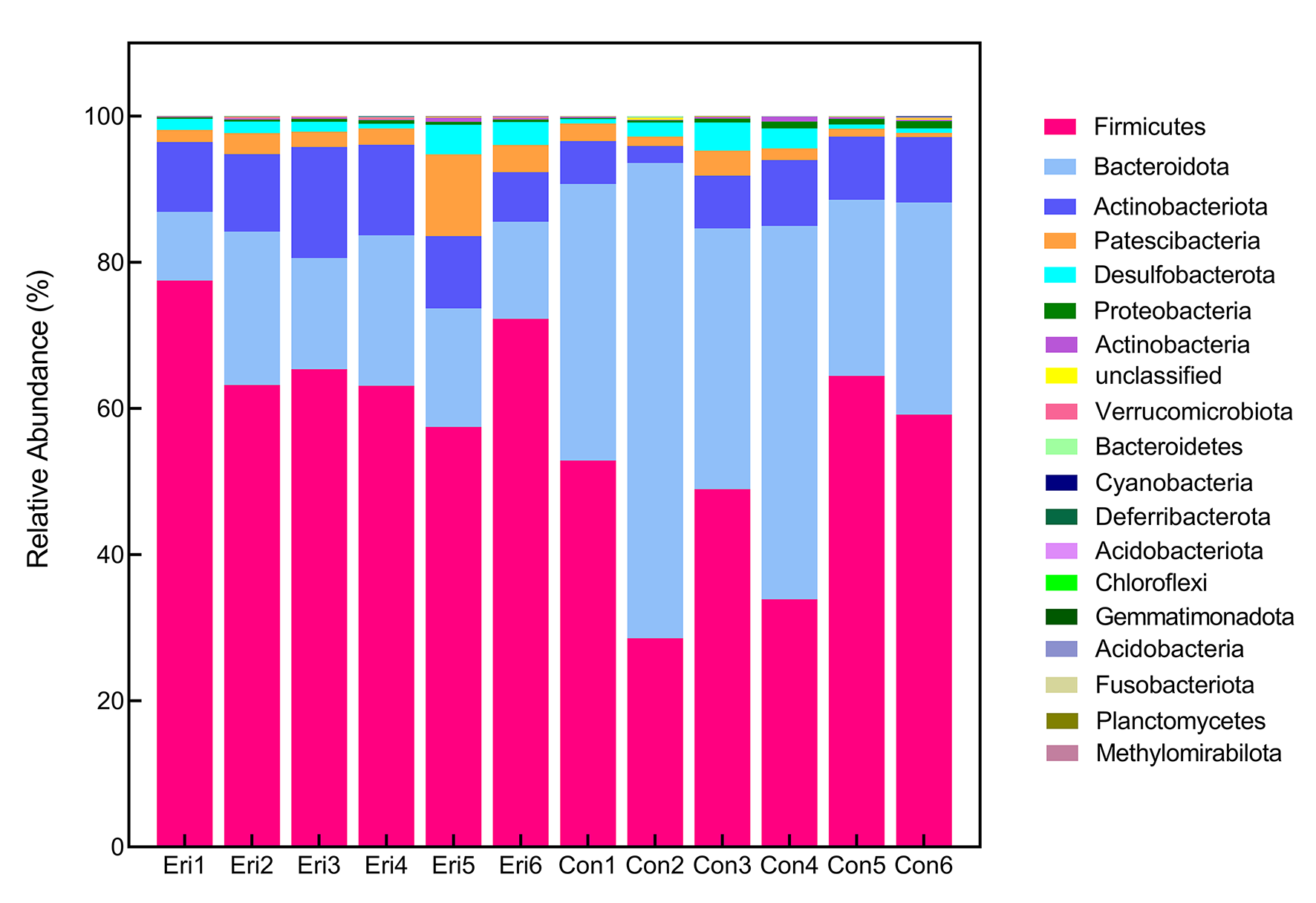

Supplement: Supplementary file 2 [file Image_1.TIF]

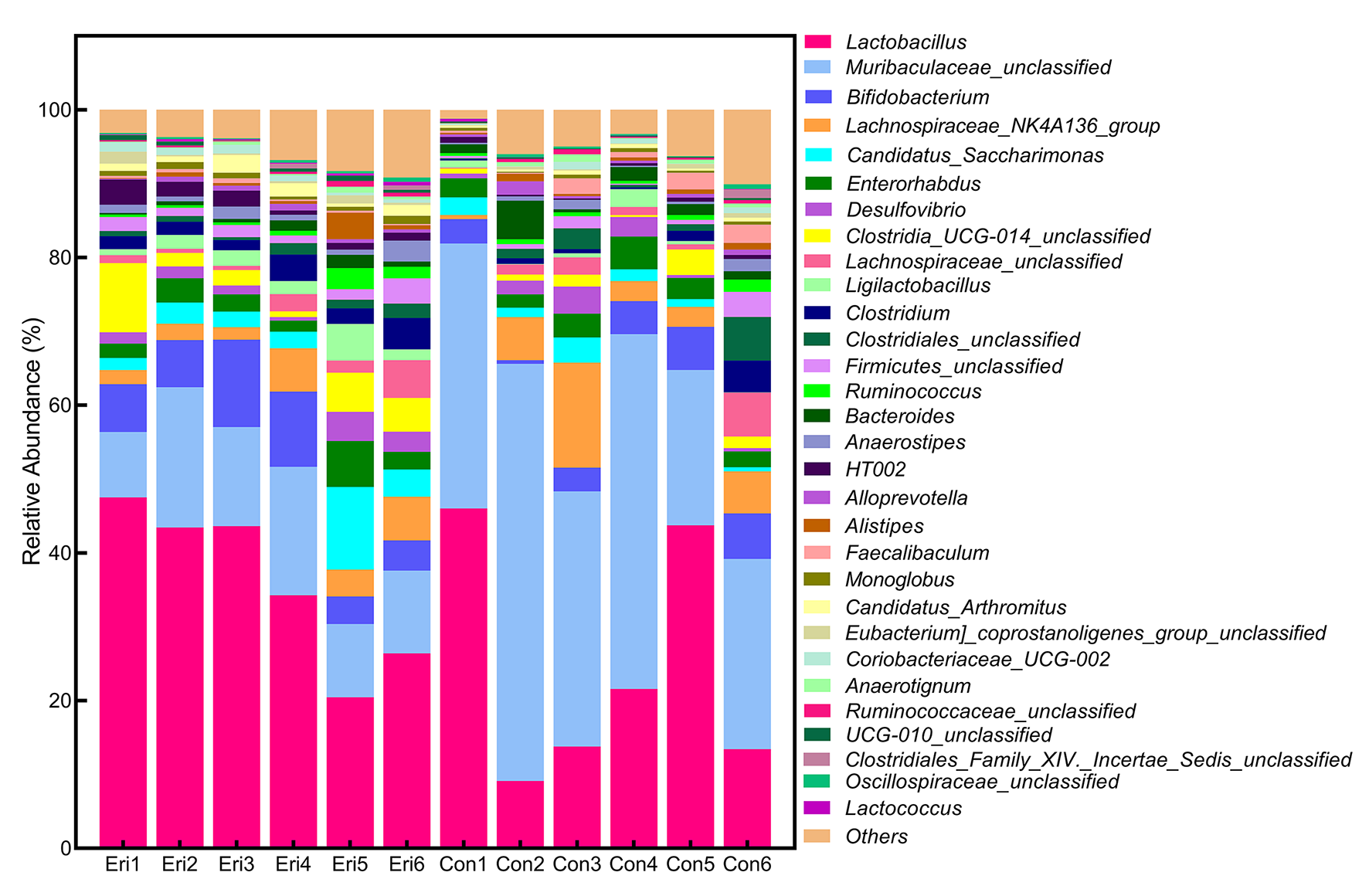

Supplement: Supplementary file 3 [file Image_2.TIF]
